# Ulcer of the Stomach

**Published:** 1848-04

**Authors:** 


### BIRMINGHAM PATHOLOGICAL SOCIETY.

*Ulcer of the Stomach: Perforation.*—October 10th, 1847. Mr. Russell was called to see, with Dr. Melson, a servant, middle aged, mother of two children, to whom Dr. Melson had been called early in the day; she had often been a free patient of Dr. Melson's, suffering from dyspepsia and gastrodynia. In the morning Dr. Melson had found her with great pain in the belly, and vomiting; abdomen much swollen and extremely tender; she had not been able to micturate. He ordered two grains of opium, four grains of calomel, and a prussic acid mixture. In the evening Dr. Melson and Mr. Russell found her in bed on her back; the vomiting had ceased, but she was in great pain; her pulse quick, but not feeble; tongue dry; abdomen very tympanitic, and extremely tender; bladder empty. At 10 P. M. she was much the same, the abdominal tenderness had increased, but the pulse was firm. She died between one and two in the night.

*Sectio Cadaveris*, forty hours after death.—The body was well nourished, and had a healthy aspect; the abdomen greatly distended; a large chamber-pot was quite filled with yellowish fluid, contained in the abdomen, in which were floating globules of castor oil, which she had taken of her own accord immediately before her death; the surface of the bowels was vascular, and thinly coated with recent lymph; the intestines perfectly healthy throughout, and without a trace of ulceration; stomach appeared healthy externally, but on being opened, a round ulcer, large enough to allow a lead pencil to pass, was found in the upper part of the anterior wall, to the left of middle line; it was clean cut, and a few fine shreds of cellular tissue still stretched across it. The mucous membrane of the organ I thought rather soft, as it did not tear off in strips, and the muscular coat was hypertrophied; the stomach offered unusual resistance to the scissors, and the middle coat was seen whiter and thicker than usual. There was a fibrous tumour, about the size of a large walnut, in the wall of the uterus.

The subject of the second case was a servant girl; she was only ill for a few hours. She had been subject to femoral hernia of the right side, but it was reducible at the time of her decease. An inquest was held on the body on account of the suddenness of the death.

The abdomen was distended with about three quarts of yellowish fluid; the surface of the intestine covered with recent lymph. The stomach was perforated by a round ulcer, about the size, internally, of the end of the little finger; externally, somewhat smaller, in the upper and left part of the anterior wall; the perforation was situated in a very thickened portion of the coats of the organ, and was about the size of a crown-piece. The stomach afforded unusual resistance to the scissors when cut; intestines healthy; hernia reduced; the liver presented a peculiar sodden appearance.

This specimen was sent to Mr. Russell by Mr. Percival, who opened the body, but could not learn more of the history of the case.

*Rupture of the Fallopian Tube.*—The following was the history of the ruptured Fallopian tube:—

Mr. Russell was sent for hastily at half-past two, P. M., to a young lady, about twenty-five, married about four months. He found her in bed, in a state of great collapse; skin cold, pale, and bloodless, and she complained of intense pain in her bowels. She had been sick, and had had three purging stools; abdomen very tympanitic and tender. She had been in this state for two hours, without having been able to procure advice. He could obtain but little information from herself as to her previous state, and could not trace her having ever suffered any local pain. She did not think herself in the family way, as she had menstruated a fortnight previously, and had not experienced any symptom which would lead her to suppose herself pregnant. Mr. Russell had little doubt that some perforation existed, and would have supposed it seated in the bowels, but that her bowels had been moved, which he never knew to occur in such cases. She referred her pain chiefly to the præcordia. Her pulse was scarcely perceptible. Fomentations, opium, and ammonia, as she objected to brandy, were ordered. She somewhat recovered, but soon sank again, and died in great pain early on the following morning.

